# Integrated Treatment of Aqueous Extract of *Solanum nigrum*-Potentiated Cisplatin- and Doxorubicin-Induced Cytotoxicity in Human Hepatocellular Carcinoma Cells

**DOI:** 10.1155/2015/675270

**Published:** 2015-06-28

**Authors:** Chien-Kai Wang, Yi-Feng Lin, Cheng-Jeng Tai, Chia-Wowi Wang, Yu-Jia Chang, Chen-Yen Choong, Chi-Shian Lin, Chen-Jei Tai, Chun-Chao Chang

**Affiliations:** ^1^Department of Obstetrics and Gynecology, School of Medicine, College of Medicine, Taipei Medical University, Taipei 110, Taiwan; ^2^Division of Hematology and Oncology, Department of Internal Medicine, Taipei Medical University Hospital, Taipei 110, Taiwan; ^3^Department of Internal Medicine, School of Medicine, College of Medicine, Taipei Medical University, Taipei 110, Taiwan; ^4^Department of Chinese Medicine, Taipei Medical University Hospital, Taipei 110, Taiwan; ^5^Division of General Surgery, Department of Surgery, Chi-Mei Medical Center, Tainan 710, Taiwan; ^6^Department of Obstetrics and Gynecology, Taipei Medical University Hospital, Taipei 110, Taiwan; ^7^Cancer Research Center, Taipei Medical University and Hospital, Taipei 110, Taiwan; ^8^Department of Surgery, Taipei Medical University and Hospital, Taipei 110, Taiwan; ^9^Division of General Surgery, Department of Surgery, Taipei Medical University Hospital, Taipei Medical University, Taipei 110, Taiwan; ^10^Graduate Institute of Clinical Medicine, College of Medicine, Taipei Medical University, Taipei 110, Taiwan; ^11^Graduate Institute of Medical Sciences, College of Medicine, Taipei Medical University, Taipei 110, Taiwan; ^12^Traditional Herbal Medicine Research Center, Taipei Medical University Hospital, Taipei 110, Taiwan; ^13^Division of Gastroenterology and Hepatology, Department of Internal Medicine, Taipei Medical University Hospital, Taipei 110, Taiwan

## Abstract

Chemotherapy is the main approach for treating advanced and recurrent hepatocellular carcinoma (HCC), but the clinical performance of chemotherapy is limited by a relatively low response rate, drug resistance, and adverse effects that severely affect the quality of life of patients. The aqueous extract of *Solanum nigrum* (AE-SN) is a crucial ingredient in some traditional Chinese medicine (TCM) formulas for treating cancer patients and exhibits antitumor effects in human HCC cells. Therefore, this study examined the tumor-suppression efficiency of AE-SN integrated with a standard chemotherapeutic drug, namely, cisplatin or doxorubicin, in human HCC cells, namely, Hep3B and HepJ5. The results suggested that the integrated treatment with AE-SN-potentiated cisplatin and doxorubicin induced cytotoxicity through the cleavage of caspase-7 and accumulation of microtubule-associated protein-1 light chain-3 A/1B II (LC-3 A/B II), which were associated with apoptotic and autophagic cell death, respectively, in both the Hep3B and HepJ5 cells. In conclusion, AE-SN can potentially be used in novel integrated chemotherapy with cisplatin or doxorubicin to treat HCC patients.

## 1. Introduction

Liver cancer is one of the most common malignant diseases worldwide, particularly in eastern Asia and sub-Saharan Africa, and hepatocellular carcinoma (HCC) is the most prevalent type of liver cancer [1]. A major challenge in treating HCC is the poor prognosis for advanced and recurrent cases. Although chemotherapy is the main approach used to treat advanced and recurrent HCC cases, its clinical performance is largely limited by various factors such as a relatively low response rate, drug resistance, and various adverse effects that substantially impact the quality of life (QOL) of HCC patients [2]. The development of complementary and alternative medicines for improving the tumor-suppression efficiency of current chemotherapeutic drugs and managing the QOL of HCC patients has become an accepted optional approach worldwide [3, 4]. Traditional Chinese medicine (TCM) has long been employed in treating various cancers through the use of numerous herb-based formulas; however, most of these formulas lack sufficient, basic clinical medical evidence verifying their antitumor efficacy.

TCM formulas are normally prepared using mixed extracts, with the composition and dose of the ingredients sometimes varying among individual cases. The varying composition and dosage cause difficulty in clarifying the antitumor efficacy of the formulas in clinical trials and experimental studies [2]. An alternative approach is examining the individual ingredients from specific TCM formulas that may contribute to the tumor-suppression efficacy. For instance, recent studies have suggested that certain crude extracts in TCM formulas, such as extracts of* Semen Coicis*,* Scutellaria barbata*, and* Solanum nigrum*, exhibited tumor-suppression efficacy in human HCC cells [5–7]. In recent studies, total flavonoids extracted from* Scutellaria barbata* inhibited cell proliferation and invasion of hepatocarcinoma via mediation of matrix metalloproteinases and metalloproteinases [8], and saikosaponin-D extracted from* Bupleurum chinense *DC also was reported to enhance radio sensitivity on hepatoma cells by adjusting cell cycle or hypoxic conditions [9, 10]. These findings suggested that components and crude extracts of some TCM herbs may inhibit hepatocarcinoma cells via various mechanisms. The crude extracts of* Solanum nigrum* have demonstrated antitumor effects in various types of cancer, including human melanoma and colorectal, endometrial, cervical, and breast cancers [11–15]. Previous studies have indicated that the aqueous extract of* Solanum nigrum* leaves (AE-SN) mainly suppressed tumor cell growth by activating programmed cell death associated with caspase-3-dependent apoptosis [7] and LC-3 A/B-related autophagy [7, 11, 12, 14]. In addition, AE-SN is capable of enhancing the cytotoxicity induced by various chemotherapeutic drugs, including cisplatin, doxorubicin, and docetaxel, in human endometrial and colorectal cancer cells [11, 12], suggesting that AE-SN is a potential ingredient to develop for integrated chemotherapy with standard chemotherapeutic drugs. Because cisplatin and doxorubicin are the standard therapeutic drugs for treating HCC cases, knowing the antitumor effects of AE-SN in combination with either cisplatin or doxorubicin in human HCC cells would be beneficial.

To understand the potential of AE-SN for use in integrated chemotherapy with cisplatin or doxorubicin in human HCC cells, the main aim of the present study was to clarify whether AE-SN enhances the cytotoxicity induced by cisplatin and doxorubicin in human HCC cells. The results showed that a single treatment with AE-SN activated programmed cell death and provides insight into the efficacy of integrating AE-SN with chemotherapeutic drugs in treating HCC cells. The study results provide experimental evidence for supporting further application of AE-SN in HCC therapy.

## 2. Materials and Methods

### 2.1. Cell Lines and Regents

Two human HCC cell lines, namely, Hep3B and HepJ5, and one normal human pulmonary fibroblast, namely, WI-38, were purchased from the Bioresource Collection and Research Center (Hsinchu, Taiwan). Hep3B is an HCC cell line commonly used to examine antitumor components, and, by comparison, HepJ5 is a more malignant and resistant cell line that exhibits high expression of survivin [16]. This study used these two HCC cell lines to examine the antitumor effects of AE-SN and used WI-38 cells to examine the cytotoxicity of AE-SN in normal human cells. All of the cell lines were maintained in Dulbecco's modified Eagle's medium (Gibco, Grand Island, NY, USA) in addition to 100 U/mL of penicillin and 100 *μ*g/mL of streptomycin (Invitrogen Life Technologies, Carlsbad, CA, USA) at 37°C in a 5% CO_2_ humidified incubator. Cisplatin and doxorubicin were purchased from Sigma-Aldrich (St. Louis, MO, USA). The cell lysis buffer was prepared using a solution of 150 mM NaCL, 50 mM Tris-HCL (pH 7.5), 1% NP-40, 0.5% deoxycholate, 0.1% SDS, 1 mM PMSF, 10 *μ*g/mL of leupeptin, and 100 *μ*g/mL of aprotinin. The primary antibodies used to detect protein expression and activation were LC-3 A/B, caspase-3, and caspase-7 (Cell Signaling Technology, Danvers, MA, USA) as well as glyceraldehyde 3-phosphate dehydrogenase (GAPDH, AbFrontier, Seoul, South Korea). A secondary antibody, donkey anti-rabbit horseradish peroxidase conjugate, was purchased from Santa Cruz Biotechnology (Santa Cruz, CA, USA). HPLC-grade acetonitrile (ACN) and methanol were purchased from JT Baker (Phillipsburg, NJ, USA). The purified solamargine was purchased from Tauto Biotech (Shanghai, China).

AE-SN was prepared using the TCM processing method to simulate the AE-SN administered to patients in TCM clinical practice. In brief, 50 g of dried* Solanum nigrum* leaves was immersed in 750 mL of distilled water, gradually heated to 100°C, and maintained at 100°C for 1 h. The AE-SN solution was further condensed to a final concentration of 1 g of raw material/mL through heating at 100°C. The final AE-SN stock solution (1 g/mL) was filtered using a 0.22 *μ*m filter before the experiment.

### 2.2. Quantitative Analysis of Solamargine in AE-SN

**Figure 1:**
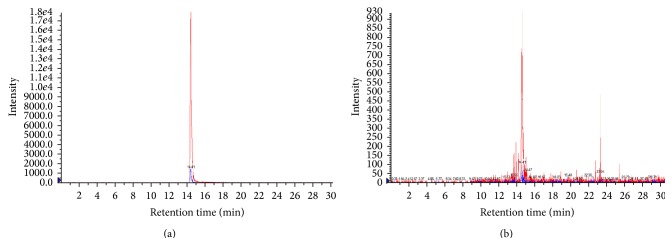
Extracted ion chromatography of solamargine. (a) The parent ion of purified solamargine was fragmented into two daughter ions (blue and red peaks). (b) Fragmented ions presented in AE-SN.

Solamargine is considered as a crucial antitumor component that existed in* Solanum nigrum* and can be a marker component for AE-SN [17]. The concentration of solamargine was therefore determined in the AE-SN stock solution by using liquid chromatography-mass spectrometry (LC-MS/MS) analysis. Before analysis, 100 *μ*L AE-SN stock solution was mixed with 900 *μ*L mixture of water : methanol (30 : 70, v/v). LC-MS/MS analysis was performed in the mobile phase of HPLC-grade acetonitrile (ACN) and deionized water with a Syncronis C18 column (Thermo Scientific, MA, USA). The flow rate is 0.5 mL/min with splitted 0.25 mL/min to mass. The ionization mode of the mass spectrometry condition was set as electrospray/positive ionization, and the mass scanning mode was multiple reaction monitor (MRM). The parent ion of purified solamargine is 867.5 *m*/*z* and fragmented into two daughter ions: 722.5 and 396.4 *m*/*z*, respectively (Figure 1(a)). These daughter ions were used to determine the solamargine concentration in AE-SN (Figure 1(b)). The concentration of solamargine in AE-SN stock solution was 77 *μ*g/mL by using MRM analysis.

### 2.3. Cell Viability Assay and Morphological Observation

Hep3B and HepJ5 cells were plated onto 96-well plates, with 5 × 10^3^ cells per well, and cultured overnight before treatment. To evaluate the antitumor effects of AE-SN, the cells were treated with 0 to 2.0 mg/mL of AE-SN for 48 h. To evaluate the antitumor effects of integrated treatment with the chemotherapeutic drugs and AE-SN, the cells were treated with 0 to 20 *μ*M cisplatin or 0 to 10 *μ*M and 0, 0.5, or 1.0 mg/mL of AE-SN for 48 h. In this study, cell viability was determined using a 3-(4,5-dimethylthiazol-2-yl)-2,5-diphenyltetrazolium bromide (MTT) assay (Table 1).

Two approaches, namely, microscopic observation and measurement of the cell size distribution, were performed to inspect the morphological changes in AE-SN-treated HCC cells. General morphological changes were observed using a Nikon Eclipse TS100 optical microscope (Nikon Instruments, Melville, NY, USA) and photographed at 100x magnification, whereas the distribution of cell diameter was measured using a Scepter cell counter (Merck Millipore Billerica, MA, USA), which divided surviving cells from cell fragments and debris in a borderline of 12 *μ*m [18].

### 2.4. Western Blotting Analysis

Hep3B and HepJ5 cells were planted into 6 cm dishes with 5 × 10^5^ cells per dish and cultured overnight. The cells were treated with a control medium, namely, 5 *μ*M of cisplatin or 2 *μ*M of doxorubicin, and 0 or 1.0 mg/mL of AE-SN for 48 h. After 48 h of incubation, the cells were collected using a cell lysis buffer. The protein concentrations of the cell lysates were determined using a Bio-Rad protein assay kit (Bio-Rad Laboratories, Hercules, CA, USA) and equalized before western blotting analysis. The cell lysates were separated using 12.5% sodium dodecyl sulfate polyacrylamide gel electrophoresis and transferred into a polyvinylidene fluoride membrane (Pall Corp, Port Washington, NY, USA).

The expression and activation of the selected protein markers, namely, LC-3 A/B, caspase-3, and caspase-7, as well as the internal control, GAPDH, were then determined using corresponding primary and secondary antibodies. The immunoreactivity was detected using an electrochemiluminescence western blotting detection kit (Western Lightning Plus-ECL, PerkinElmer Inc., Waltham, MA, USA).

### 2.5. Statistical Analysis

Analyses of the half maximum inhibitory concentration (IC_50_) were performed using CalcuSyn software (Biosoft, Cambridge, UK), whereas Student's *t* test and one-way ANOVA were performed using SPSS software (SPSS Inc., Chicago, IL, USA).

## 3. Results

### 3.1. Cytotoxicity of AE-SN Alone and with the Chemotherapeutic Drugs in Hep3B and Hep5J Cells

**Figure 2:**
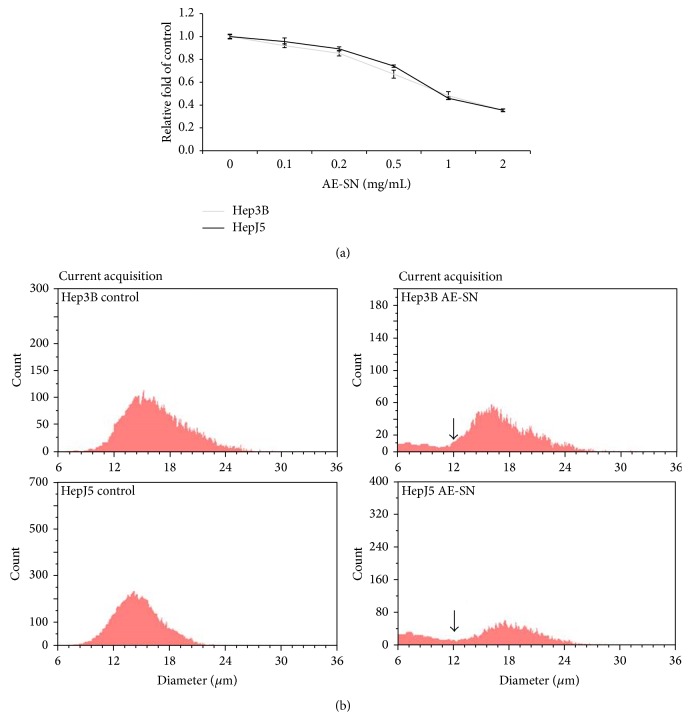
AE-SN treatment inhibited Hep3B and HepJ5 cell growth. (a) Hep3B and HepJ5 cells were treated with 0.1 to 2 mg/mL of AE-SN for 48 h, and the cell viability was determined using an MTT assay. The data are presented as the mean ± standard deviation. (b) Hep3B and HepJ5 cells were treated with 1.0 mg/mL of AE-SN for 48 h, and the cell size distribution was determined according to the cell diameter by using a Scepter cell counter. Arrows indicate a cell diameter of 12 *μ*m.

In this study, 48 h treatment with 0 to 2.0 mg/mL of AE-SN gradually inhibited the growth of Hep3B and HepJ5 cells in a concentration-dependent manner (one-way ANOVA, *P* < 0.01, Figure 2(a)). The IC_50_ values of AE-SN for the Hep3B and HepJ5 cells were 0.96 and 0.97 mg/mL, respectively. AE-SN treatment also resulted in the production of cell fragments and debris, which were less than 12 *μ*m in diameter in comparison with cells treated with the control medium in both Hep3B and HepJ5 cells (Figure 2(b)). Collectively, these results suggested that AE-SN inhibited cell growth and demonstrated cytotoxicity in Hep3B and HepJ5 cells.

**Figure 3:**
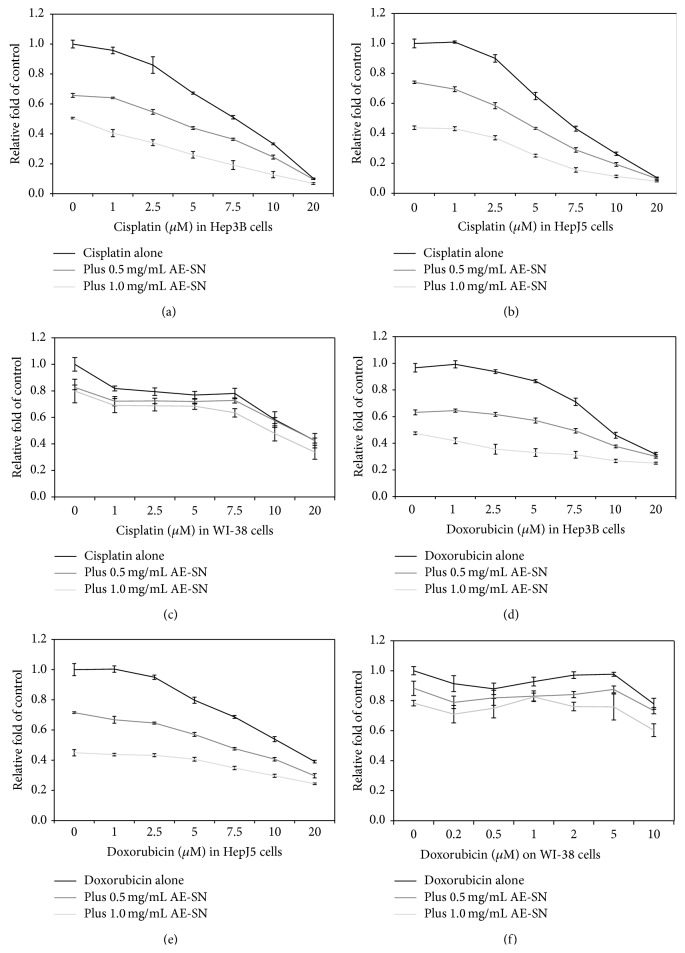
AE-SN-potentiated cisplatin and doxorubicin induced cytotoxicity in Hep3B and HepJ5 cells but had no effect on normal human pulmonary fibroblasts (WI-38 cells). (a–c) Cells were treated with 0 to 20 *μ*M cisplatin and 0, 0.5, or 1.0 mg/mL of AE-SN for 48 h. (d–f) Cells were treated with 0 to 10 *μ*M doxorubicin and 0, 0.5, or 1.0 mg/mL of AE-SN for 48 h. The cell viability was determined using an MTT assay; the data are presented as the mean ± standard deviation.

To evaluate cytotoxicity on the integrated treatment with chemotherapeutic drugs and AE-SN, cisplatin (1 to 20 *μ*M) or doxorubicin (1 to 10 *μ*M) was used to treat the Hep3B and HepJ5 cells, respectively, with 0, 0.5, or 1.0 mg/mL of AE-SN for 48 h (Figure 3). The IC_50_ values of the cisplatin integrated with 0.5 mg/mL of AE-SN in the Hep3B and HepJ5 cells were reduced to 2.74 *μ*M and 2.84 *μ*M, respectively, whereas those of doxorubicin were reduced to 1.31 *μ*M and 1.42 *μ*M. In other words, the IC_50_ values for cisplatin and doxorubicin integrated with 0.5 mg/mL of AE-SN were reduced to 40% and 30% of the values of the drugs used alone in the Hep3B and J5 cells, respectively. Human pulmonary fibroblast cells, WI-38, were also treated with 0, 0.5, or 1.0 mg/mL of AE-SN and cisplatin or doxorubicin to identify the cytotoxicity of the combined treatment (Figures 3(c) and 3(f)). AE-SN was not likely to enhance cisplatin- or doxorubicin-induced cytotoxicity in the WI-38 cells in comparison with that in the Hep3B and HepJ5 cells. Together, these results suggested that the AE-SN treatment potentiated cisplatin- and doxorubicin-induced cytotoxicity in both Hep3B and HepJ5 cells but not in WI-38 normal human cells.

### 3.2. Activation of Programmed-Cell-Death-Related Protein in AE-SN and Chemotherapeutic-Drug-Treated Cancer Cells

**Figure 4:**
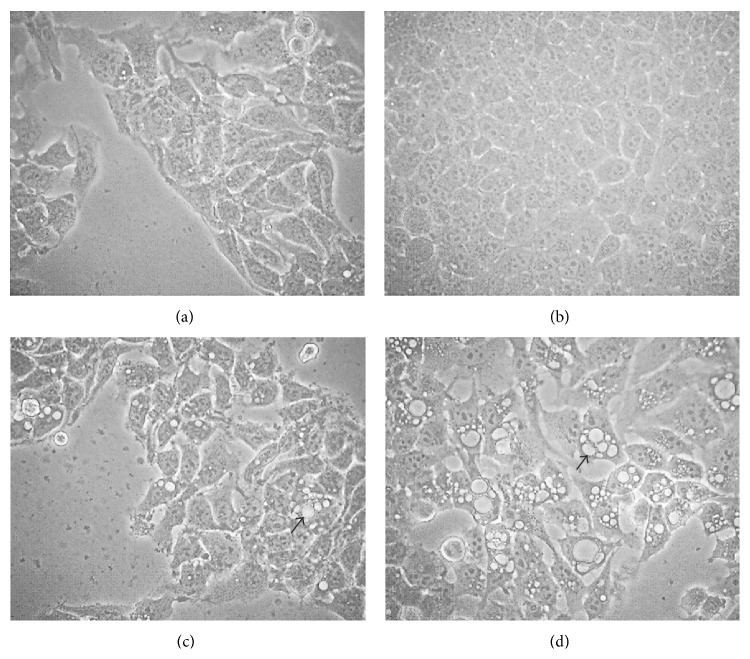
AE-SN-activated programmed cell death in Hep3B and HepJ5 cells. (a) Hep3B and (b) HepJ5 cells were treated with a control medium for 48 h. (c) Hep3B and (d) HepJ5 cells were treated with 1.0 mg/mL of AE-SN for 48 h. Arrows indicate the morphological changes present in the AE-SN-treated cells (100x magnification).

Hep3B and HepJ5 cells treated with AE-SN demonstrated a clear morphological change similar to the formation of phagolysosome-like vacuoles (Figures 4(a) to 4(d)). This morphological change was also observed in AE-SN-treated human endometrial and colorectal carcinoma cells [11] and related to the activation of autophagy.

**Figure 5:**
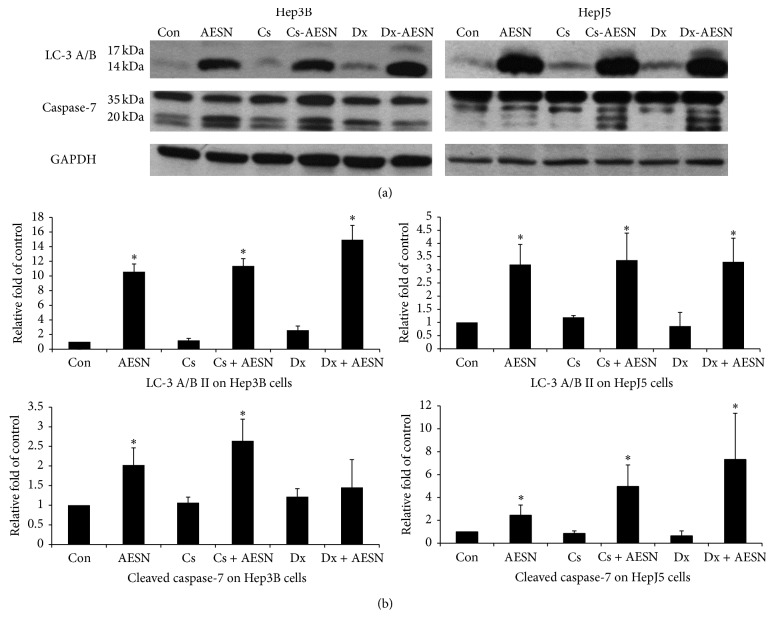
Activation of selected protein markers in HCC cells treated with AE-SN and either cisplatin or doxorubicin. (a) Cells were treated with a control medium, namely, 5 *μ*M cisplatin or 2 *μ*M doxorubicin with 0 or 1.0 mg/mL of AE-SN for 48 h. The activation of LC-3 A/B and caspase-7 was determined using western blotting analysis. GAPDH served as an internal control. (b) Semiquantitation of the LC-3 A/B II and cleaved caspase-7 in the Hep3B and HepJ5 cells. The data are presented as the mean ± standard deviation. *∗* indicates statistical significance in comparison with the previous group, namely, Con versus AESN (vehicle control versus AE-SN), Cs versus Cs + AESN, and Dx versus Dx + AESN (two-tailed Student's *t*-test, *P* < 0.05).

To further understand whether AE-SN increases the activation of cell death protein when combined with either cisplatin or doxorubicin in the two HCC cell lines, the activation of LC-3 A/B and caspase-7 was observed in HCC cells treated with 1 mg/mL of AE-SN and 5 *μ*M cisplatin or 2 *μ*M doxorubicin for 48 h. As shown in Figure 5, the results suggested that, in both Hep3B and HepJ5 cells, AE-SN induced LC-3 A/B II accumulation in all of the treatment groups. The cleavage of caspase-7 was also enhanced by AE-SN cotreatment in cisplatin-treated Hep3B and HepJ5 cells and in doxorubicin-treated HepJ5 cells, but not in doxorubicin-treated Hep3B cells. Doxorubicin cotreatment seemed to eliminate the AE-SN-induced caspase-7 cleavage in the Hep3B cells.

## 4. Discussion

In this study, the impact of AE-SN on tumor-suppression efficiency was evaluated by employing the preparation of AE-SN that is used in TCM clinical practice. The IC_50_ values of AE-SN for Hep3B and HepJ5 cells indicated a moderate antitumor effect through direct exposure. Our previous studies showed that administering AE-SN at approximately 0.5 to 1 mg/mL for 48 h yielded similar IC_50_ values in human endometrial and colorectal cancer cells [11, 12]. In comparison with the tumor-suppression efficiency observed in previous studies using AE-SN extracts prepared from dried powder through water extraction [7, 13, 14, 19, 20], the tumor-suppression efficiency of AE-SN in this study was similar. The results of these experimental studies collectively suggest that cancer treatment may be a reasonable application for the preparation of AE-SN used in TCM practice. In addition, integrated treatment of AE-SN with cisplatin and doxorubicin may substantially reduce the required dose of cisplatin and doxorubicin required to achieve the same tumor-suppression efficiency, thereby improving the QOL of HCC patients during chemotherapy.

AE-SN was suggested to activate both autophagic and apoptotic cell death in many human cancer cell lines by inducing the cleavage of caspase-3 and accumulation of LC-3 A/B II [7]. By contrast, this study indicated that AE-SN treatment failed to induce the cleavage of caspase-3 in both Hep3B and HepJ5 cells (data not shown). This result coincided with observations of human colorectal and endometrial cancer cells [11, 12] that suggested that resistance to AE-SN-induced caspase-3 cleavage may vary among cancer cell types. Cleaved caspase-7 is another biomarker for apoptotic cell death and was found to be induced in Hep3B and HepJ5 cancer cells after a single treatment with AE-SN. The cleavage of caspase-7 can be induced through mitochondria-mediated and extracellular signal-induced apoptosis pathways [21]. AE-SN-induced caspase-7 cleavage may occur through an alternative signal pathway, independent of caspase-3 cleavage, and activates apoptosis in caspase-3 resistant cancer cells. In addition, AE-SN induces LC-3 A/B II accumulation, which is associated with autophagy activation in Hep3B and HepJ5 cells. These results have been a common feature among AE-SN studies [7, 11, 12, 14]. In integrated treatment, AE-SN still induced both LC-3 A/B II accumulation and caspase-7 cleavage in both cisplatin- and doxorubicin-treated cells, suggesting that AE-SN may enhance cisplatin- and doxorubicin-induced cytotoxicity through the activation of autophagy and caspase-7-related apoptosis.

In comparison with Hep3B cells, HepJ5 cells are more malignant and resistant and exhibit a higher expression of survivin [16]. In the present study, the IC_50_ values for cisplatin and doxorubicin in HepJ5 cells were higher than those in Hep3B cells (8.71 versus 6.75 and 6.39 versus 4.65 *μ*M, resp.). This result confirmed that HepJ5 cells are more resistant to cisplatin- and doxorubicin-induced cytotoxicity than Hep3B cells were. By contrast, the IC_50_ values for AE-SN were similar in HepJ5 and Hep3B cells (0.97 versus 0.96 mg/mL), and combined treatment with cisplatin or doxorubicin and 0.5 mg/mL of AE-SN further reduced the IC_50_ values to the same levels (2.84 versus 2.74 *µ*M in the cisplatin combination and 1.31 versus 1.42 *µ*M in the doxorubicin combination). These results collectively suggested that HepJ5 cells were unable to resist AE-SN-induced cytotoxicity and tended to be more vulnerable to cisplatin- and doxorubicin-induced cytotoxicity in combined treatment with AE-SN. The molecular mechanisms involved in this AE-SN-mediated HCC cytotoxicity require further investigation.

Some steroidal alkaloid glycosides and glycoproteins are considered the major active substances responsible for AE-SN-induced programmed cell death. Solamargine is one of the major compounds present in AE-SN that can activate caspase-3-related apoptosis and LC-3-A/B-II-related autophagy in human leukemia cells [22], and a 150 kDa glycoprotein isolated from AE-SN was reported to be another effective antitumor compound for activating caspase-3-related apoptosis in human colorectal, cervical, and HCC cells [23–25]. However, these isolated compounds do not seem to completely explain the tumor-suppression mechanism of AE-SN because the specific compound that activates the cleavage of caspase-7 remains unknown. Further investigation is therefore required to identify the exact composition of the AE-SN substance that contributes to the antitumor effects.

This study clarified the antitumor effects of AE-SN and the potential of AE-SN for enhancing cytotoxicity induced by cisplatin and doxorubicin in human HCC cells in vitro. In consideration of the absorption rate of AE-SN through the gastrointestinal tracts and its metabolism in vivo, the real tumor-suppression efficiency of AE-SN with chemotherapeutic drugs as well as the optimal dosage, preparation, and administrative approach of AE-SN remains to be evaluated using an animal cancer model. In addition, any unexpected adverse effects of integrated treatment with AE-SN and chemotherapeutic drugs in vivo should be carefully examined before clinical trials are conducted.

## 5. Conclusion

**Figure 6:**
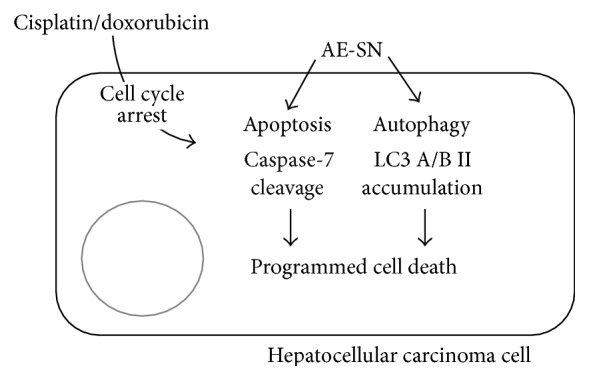
Illustration of the integrated-cell-death mechanism activated by AE-SN and either cisplatin or doxorubicin in HCC cells.

Clear experimental evidence obtained in this study indicates that the AE-SN cotreatment potentiated cisplatin- and doxorubicin-induced cytotoxicity in human HCC cells. This AE-SN-potentiated cytotoxicity may occur through the accumulation of LC-3 A/B II and cleavage of caspase-7 to activate apoptosis and autophagic cell death in human HCC cells (Figure 6). Collectively, our results suggest that AE-SN can be used in novel integrated chemotherapy with cisplatin and doxorubicin to improve tumor-suppression efficiency in HCC treatment.

## Figures and Tables

**Table 1:** IC_50_ values in HCC cells treated with AE-SN alone or in combination with AE-SN and either chemotherapeutic drug. The IC_50_ values were analyzed using cell viability data determined after 48 h treatment with AE-SN (Figure 2) and with AE-SN integrated with either chemotherapeutic drug (Figure 3).

	Hep3B		Hep5J	

AE-SN (mg/mL)	0.96		0.97	

	Combination of AE-SN (mg/mL)			
	0	0.5	0	0.5

Cisplatin (*μ*M)	6.75	2.74	8.71	2.84
Doxorubicin (*μ*M)	4.65	1.31	6.39	1.42
